# Supplementary material for: Identification of photoperiod-induced specific miRNAs in the adrenal glands of Sunite sheep (Ovis aries)
Source: Front Vet Sci. 2022 Jul 22;9:888207. doi: 10.3389/fvets.2022.888207 (PMC9354845; doi:10.3389/fvets.2022.888207)
Supplement: Supplementary file 1 [file Data_Sheet_1.ZIP › Supplementary file/miRDeep2 file.zip!/miRDeep2 file/Know_Structure/SPLP42A2/oar-let-7i.pdf]

miRBase precursor : oar-let-7i
Total read count : 465934
oar-let-7i read count : 465711
remaining reads : 223

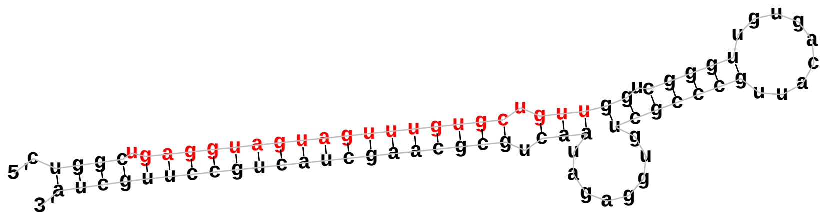

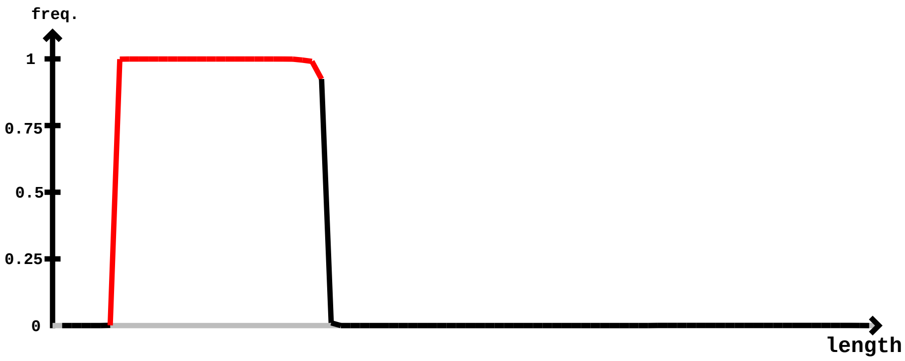


```
        oar-let-7i
5'- cuggcugagguaguaguuugugcuguuggucggguugugacauugcccgcuguggagauaacugcgcaagcuacugccuugcua  -3'    exp
    .((((.(((((((((((((((((.(((((.(((((.........))))))).........))).)))))))))))))))))))))    reads   mm   sample
    ...gcugagguaguaguuugugcu............................................................    1       0    seq
    ....cugagguaguaguuugu...............................................................    2       0    seq
    ....cugagguaguaguuugugcu............................................................    2       0    seq
    ....cugagguaguaguuugugcug...........................................................    1       0    seq
    ....cugagguaguaguuugugcugu..........................................................    24      0    seq
    ....cugagguaguaguuugugcuguu.........................................................    384     0    seq
    ....cugagguaguaguuugugcuguug........................................................    4       0    seq
    .....ugagguaguaguuugug..............................................................    37      0    seq
    .....ugagguaguaguuugugc.............................................................    121     0    seq
    .....ugagguaguaguuugugA.............................................................    13      1    seq
    .....ugagguaguaguuugugG.............................................................    4       1    seq
    .....ugagguaguaguuugugcu............................................................    1685    0    seq
    .....ugagguaguaguuugugcug...........................................................    2291    0    seq
    .....ugagguaguaguuugugcugu..........................................................    30864   0    seq
    .....ugagguaguaguuugugcuguu.........................................................    425282  0    seq
    .....ugagguaguaguuugugcuguug........................................................    4729    0    seq
    .....ugagguaguaguuugugcuguugg.......................................................    19      0    seq
    ......gagguaguaguuugugcu............................................................    4       0    seq
    ......gagguaguaguuugugcug...........................................................    4       0    seq
    ......gagguaguaguuugugcugu..........................................................    15      0    seq
    ......gagguaguaguuugugcuguu.........................................................    219     0    seq
    ......gagguaguaguuugugcuguug........................................................    2       0    seq
    .......agguaguaguuugugcuguu.........................................................    3       0    seq
    .........guaguaguuugugcuguu.........................................................    1       0    seq
    .............................................................cugcgcaagcuacugccuu....    1       0    seq
    .............................................................cugcgcaagcuacugccuug...    8       0    seq
    .............................................................cugcgcaagcuacugccuugc..    71      0    seq
    .............................................................cugcgcaagcuacugccuugcu.    143     0    seq
```
